# A Self-Powered, Threshold-Based Wireless Sensor for the Detection of Floor Vibrations

**DOI:** 10.3390/s18124276

**Published:** 2018-12-05

**Authors:** Byung C. Jung, Young Cheol Huh, Jin-Woo Park

**Affiliations:** Department of System Dynamics, Korea Institute of Machinery and Materials, Daejeon 34103, Korea; ychuh@kimm.re.kr (Y.C.H.); jwp@kimm.re.kr (J.-W.P.)

**Keywords:** vibration-based energy harvester, piezoelectric energy harvester, detection of floor vibration, smart building

## Abstract

Smart buildings will soon be a reality due to innovative Internet of Things (IoT) applications. IoT applications can be employed not only for energy management in a building, but also for solving emerging social issues, such as inter-floor noise-related disputes in apartments and the solitary death of an elderly person. For example, acceleration sensors can be used to detect abnormal floor vibrations, such as large vibrations due to jumping children or unusual vibrations in a house where an elderly person is living alone. However, the installation of a conventional accelerometer can be restricted because of the sense of privacy invasion. In this study, a self-powered wireless sensor using a threshold-based method is studied for the detection of floor vibrations. Vibration levels of a bare slab in a testbed are first measured when a slab is impacted by a bang machine and an impact ball. Second, a piezoelectric energy harvester using slab vibration is manufactured to generate electrical power over a threshold. Next, the correlation among harvested energy, floor vibration, and impact noise is studied to check whether harvested energy can be employed as a condition detection threshold. Finally, a prototype of a self-powered wireless sensor to detect abnormal conditions in floor vibrations is developed and its applicability is demonstrated.

## 1. Introduction

For the successful realization of a Smart Building [1,2], connected wireless sensors are required to detect abnormal changes in a building and make smart decisions for residents. In recent years, Internet of Things (IoT) applications with energy management technologies for building automation [3,4] have been widely developed. In addition, with the increasing urbanization and individualism of a society, studies on monitoring systems to resolve social issues, such as noise-related disputes in apartments and the solitary death of an elderly person living alone, have also been performed [5,6,7,8,9]. Lam et al. [5] developed an occupant detection method by monitoring and analyzing step-induced structural vibration in an apartment. Mun et al. [6] researched the relationships among impact force, the acceleration response of a slab, and floor impact noise. Acceleration sensors are essential to those applications; however, in some cases, privacy invasion and cyber security problems can restrict the usage of conventional sensors. For example, floor noise-related disputes in apartments have increased over the past few years and became a crucial social issue in Asian countries. Monitoring of acceleration levels with conventional accelerometers installed on floors of an apartment can solve this to support a mediation process by providing objective data on human-induced floor vibrations. However, the realization of it may not be feasible since vibration signals being measured in real-time during the day and night can be seen as privacy invasion. For example, residence patterns analyzed with real-time signals can be used in crime investigations, especially when a monitoring system is developed with a wireless system.

Energy harvesting that converts ambient, otherwise wasted, energy sources into usable electricity has been widely researched as a promising solution for wireless sensing nodes. Among the various energy harvesting devices, a piezoelectric energy harvester that generates electricity from mechanical strain energy has received much attention as a power source of wireless sensors due to its high power density and ease of installation [10,11,12,13]. When a piezoelectric material is deformed by an external mechanical force, it generates a charge flow due to the change in electric polarization. This phenomenon is called the piezoelectric effect [14,15]. A typical piezoelectric energy harvester is a cantilever-type unimorph/bimorph using a ‘31’ mode (where mechanical strain is perpendicular to the poling direction). This generates AC voltage proportional to the bending strains of a piezoelectric material [16,17,18,19], and is connected to interface electrical circuits designed by an impedance matching theory to extract maximum harvesting power [20,21,22].

**Figure 1:**
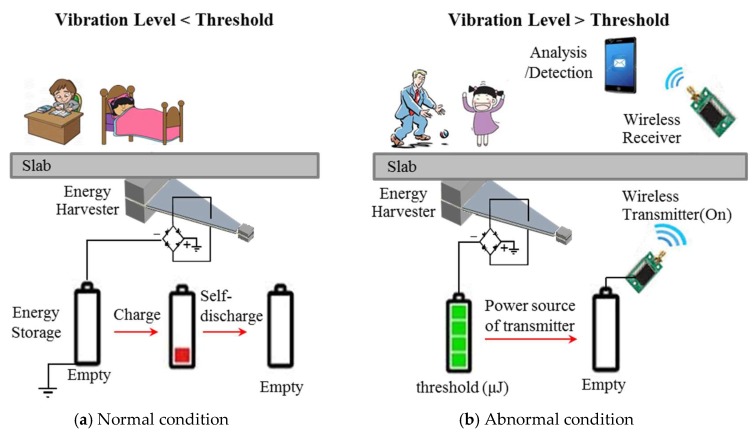
Concept of the self-powered wireless sensor for threshold-based detection of floor vibrations.

In this paper, a self-powered, threshold-based wireless sensor is proposed to detect abnormal floor vibration conditions. A cantilever-type piezoelectric energy harvester is employed to generate electric energy proportional to the amount of mechanical floor vibration. One benefit of employing an energy harvester is that accumulated electric energy can be used as a power source for a wireless data transmitter. Figure 1 shows the concept of the proposed self-powered wireless sensor. When the vibration of a slab is below a designated threshold, the amount of harvested electric energy is not high enough to operate a wireless transmitter; thus, the wireless transmitter is not activated. However, when the vibration of a slab is high enough to generate electric energy above that threshold, the wireless transmitter is activated and transmits an alarm signal to a receiver. The proposed self-powered wireless sensor has the following advantages: (1) It is relatively free from invasion of privacy, since it does not collect and save vibration signals in real-time, and only collects data during abnormal conditions; (2) there is no maintenance cost to change chemical batteries; and (3) installation is easy and inexpensive compared to wired systems that require wiring for data transmission and power supply.

The rest of this article is organized as follows. Section 2 provides a description of developing a cantilever-type piezoelectric energy harvester designed based on the dynamic characteristics of a slab in a testbed. Section 3 shows correlation studies among slab vibrations, floor impact sound, and harvested electric energy. A strong correlation among these factors is an essential requirement for indirectly detecting floor vibrations and the corresponding impact noise with harvested electric energy. Section 4 describes the integration of the energy harvester with a wireless transmission system, and gives a demonstration of the self-powered wireless sensor for the detection of floor vibrations.

## 2. Development of a Cantilever-Type Piezoelectric Energy Harvester

### 2.1. Testbed and Dynamic Characteristics of the Slab

**Figure 2:**
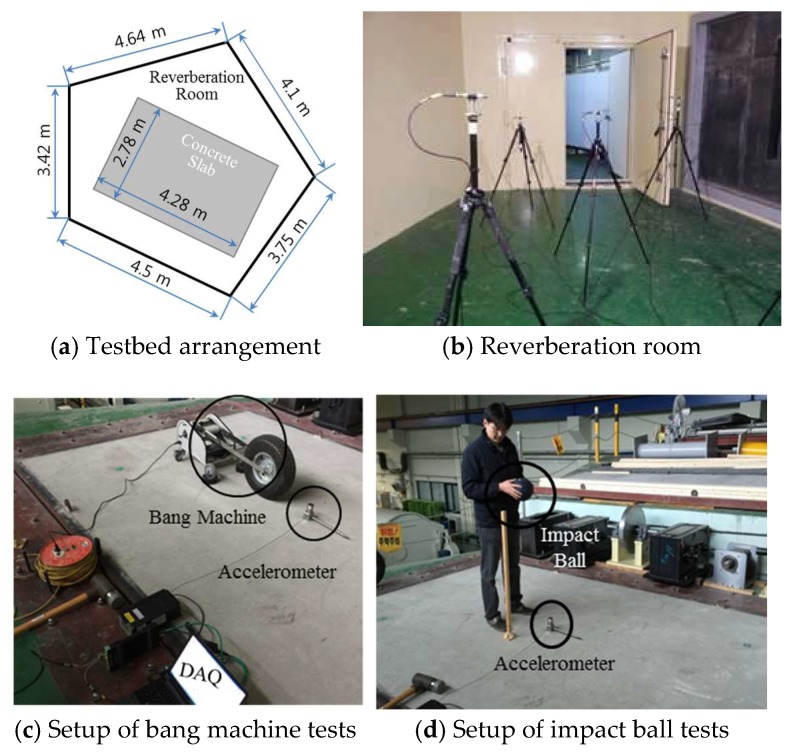
Testbed configuration and test setup.

Before designing a piezoelectric energy harvester, the dynamic characteristics of the slab in the testbed are experimentally analyzed. The testbed consists of a reverberation room and a concrete slab located on top of the reverberation room. The shape and size of the reverberation room are shown in Figure 2a. Figure 2b shows the reverberation room for measuring impact noise. Figure 2c,d show the concrete slab, and the thickness of the slab is 150 mm.

**Figure 3:**
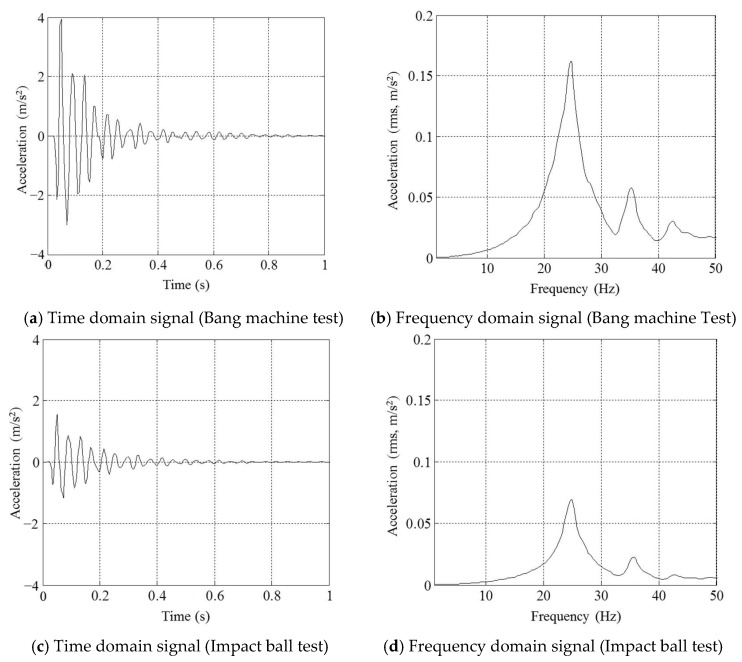
Measured acceleration signals.

Standard heavyweight impact sources (a bang machine and an impact ball) that are officially used for measuring floor impact sound are employed to excite the concrete slab. Figure 2c,d show the setups of the bang machine and impact ball tests. While impacting the center of the bare slab with the bang machine and the impact ball, vibration signals are measured with an accelerometer attached on the center of the slab. Figure 3a–d show the measured acceleration signals in time and frequency domains. 

As shown in Figure 3b,d, the natural frequency of the slab is 24.8 Hz. During the bang machine test, the maximum amplitude and time duration of the acceleration signal are about 4.0 m/s^2^ and 0.8 s, respectively. With regard to the impact ball test, the maximum amplitude and time duration of the acceleration signal are about 1.55 m/s^2^ and 0.6 s, respectively. The impact sources and dynamic characteristics of the slab are summarized in Table 1.

### 2.2. Design of the Cantilever-Type Piezoelectric Energy Harvester

**Figure 4:**
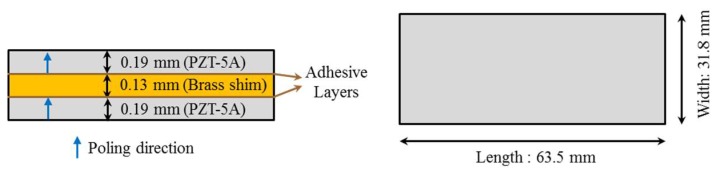
Shape and size of the base bimorph specimen.

A piezoelectric energy harvester is designed to produce the required energy (threshold) at a given impact condition. For wireless data transmission, this study employs data transmitting/receiving nodes, AmbioMote24 (Type A) from Ambiosystems, Heuvelton, NY, USA [23]. The data transmitting/receiving nodes require 5.3 V for initiating, 2.7 V for data transmitting, and about 15 μW for continuous data monitoring [10]. By considering the first resonance frequency of the slab and the power generation capacity (several tens of microwatt for wireless data transmission), a piezoelectric bimorph specimen from Piezo System Inc., Cambridge, MA, USA [24] of a 63.5 mm length and a 31.8 mm width is selected. Figure 4 and Table 2 show the shape and specification of the base bimorph specimen. The specimen consists of two piezoelectric plates with nickel electrode layers, a center brass shim, and two adhesive layers.

**Figure 5:**
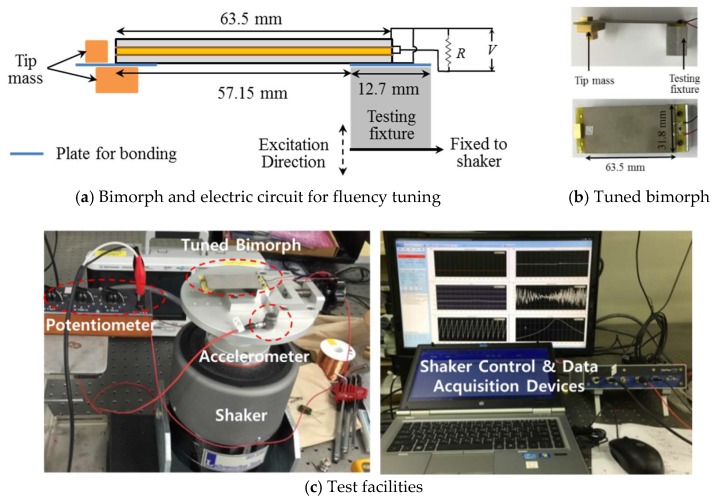
Test setup for frequency tuning.

**Figure 6:**
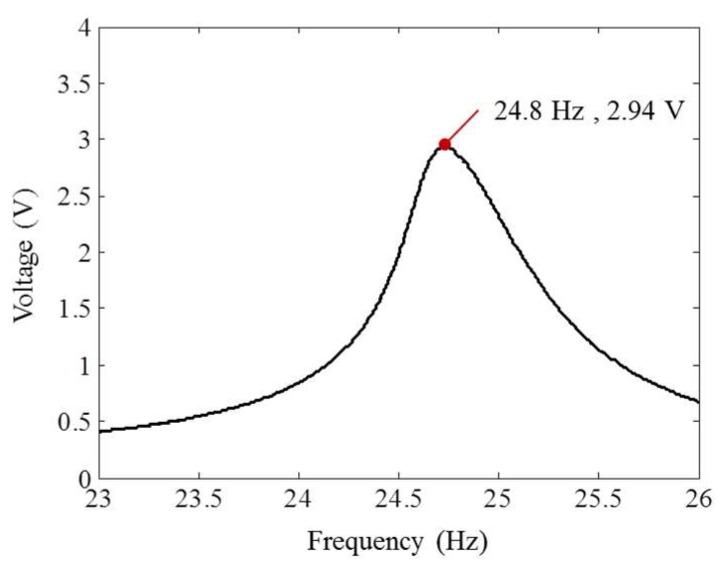
Sine sweep test results of the bimorph.

Next, the base bimorph specimen in Figure 4 is tuned to match a resonance frequency with the natural frequency of the slab. Figure 5 shows the configuration of the test setup for frequency tuning. The base specimen is fixed to a shaker (ET-139, Labworks Inc., Novato, CA, USA) using a testing fixture. The shaker is controlled with a controller (M + P Vibcontrol, m + p international, Inc., Verona, NJ, USA) and an accelerometer (3055B2T, Dytran Instruments, Chatsworth, CA, USA) in Figure 5c. A plastic plate of a 12.7 mm length is used to bond tip masses, as shown in Figure 5a. For the voltage monitoring, two wires from the specimen are connected to a resistor. The resistance of a resistor (R) is set to 10 MΩ. Sine sweep vibration tests with a 0.01 g acceleration peak are performed by increasing tip masses while measuring the voltage (V) on a resistor. The total weight of the tip masses to make the first natural frequency of the bimorph 24.8 Hz (the first natural frequency of the slab) is 15.14 g. Figure 5b shows the tuned bimorph, and Figure 6 shows the sine sweep vibration test result. At the resonant frequency, the maximum voltage of the tuned bimorph is 2.94 V.

## 3. Correlation Study

**Figure 7:**
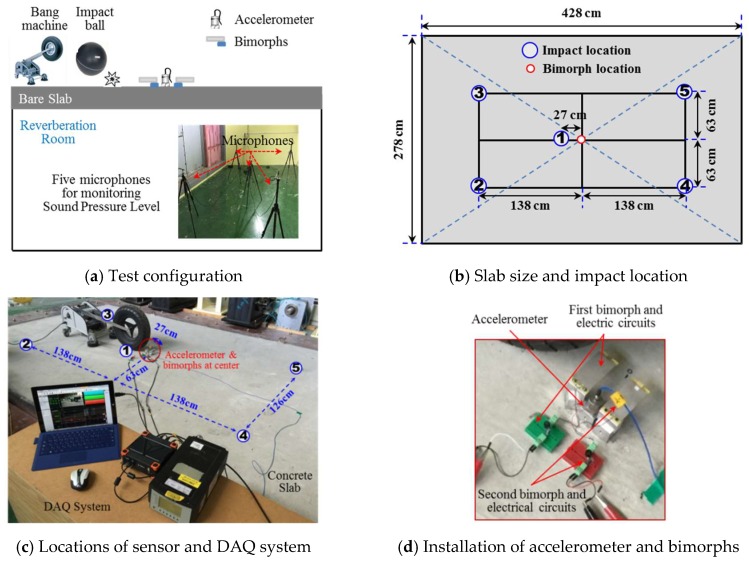
Test setup for correlation study.

The correlation among floor impact sound, slab vibrations, and harvested electric energy is studied to confirm whether harvested electric energy can be used as a quantitative threshold to predict floor vibrations and the corresponding impact sound in an apartment. As shown in Figure 7a, an accelerometer (Type 4370, B&K, Nærum, Denmark) is attached on the center of the concrete slab to measure the floor acceleration of the slab. The sound pressure level (SPL) in the reverberation room is also measured using five microphones (Tpye 4942-A-021, B&K, Nærum, Denmark), as shown in Figure 7a. As a data acquisition system, this study used SIRIUS Mini (DEWESoft, Trbovlje, Slovenia) to measure voltages at capacitors and acceleration levels. PULSE 3560C (B&K, Nærum, Denmark) is also used to measure sound pressure levels. Five locations shown in Figure 7b with circled numbers are impacted with a bang machine and an impact ball, sequentially. Thus, a total of 10 tests are performed and measured signals are analyzed. Location No. ① is 27 cm away from the center of the slab to avoid interference with the accelerometer.

**Figure 8:**
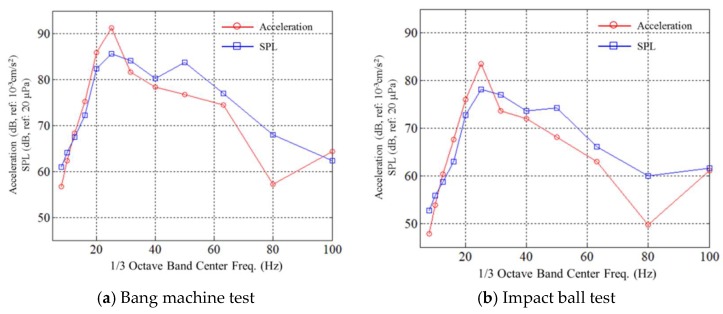
Measured acceleration and sound pressure levels.

Figure 8 shows plots of slab acceleration and sound pressure level, when location No. ① is impacted. The *X*-axis refers to the center frequencies of one-third octave bands, and the *Y*-axis indicates accelerations and sound pressure levels in decibels. The reference values are 10^−3^ cm/s^2^ and 20 μPa for acceleration and sound pressure, respectively. A clear correlation is found at the first natural frequency of the slab (≈25 Hz). Except for some frequencies where the sound pressure is amplified due to room modes, the correlation between slab acceleration and sound pressure levels is generally maintained. Measured acceleration and SPL levels are summarized in Table 3.

**Figure 9:**
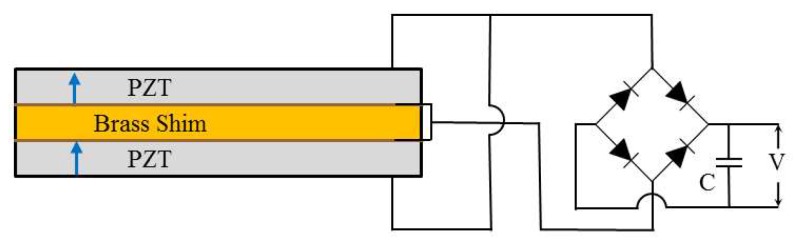
Configuration of electric circuits.

Next, two tuned bimorphs bonded on the center of the slab are employed to measure the amount of harvested electric energy from slab vibrations in Figure 7d. Two tuned bimorphs are separately connected to electric energy storage circuits. In this study, the Standard Energy Harvesting (SEH) interface circuit in Figure 9 is employed. Two wires from each bimorph are linked to a bridge rectifier and a capacitor. While impacting the slab with a bang machine and impact ball, voltage changes at capacitors are measured. The harvested energy of one bimorph is calculated using Equation (1):

Harvested Energy = 0.5·C·(V^2^ − V_0_^2^), (1)

where V_0_ and V refer to the measured voltage before and after excitation, respectively; and C refers to the capacitance of the capacitor. The capacitance (C) of the capacitor is set to 47 μF in this study. The calculated harvested energies of the two bimorphs are finally averaged, as shown in the third column in Table 3. It is known that a harvestable power (P) of a cantilever-type piezoelectric energy harvester is proportional to the square of acceleration amplitude (A_in_). Roundy et al. [10] developed an analytical equation for a two-layer bimorph to predict the harvestable power based on one-dimensional beam theory, and prove the relationship (P ∝ A_in_^2^). Thus, the square root of harvested electric energy is also calculated, as shown in the fourth column in Table 3.

**Figure 10:**
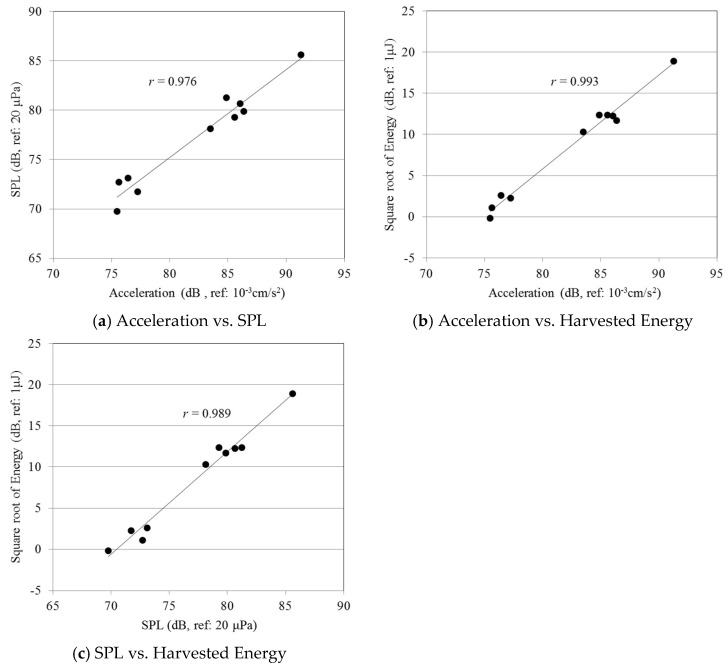
Correlation among slab acceleration, SPL, and harvested energy.

Figure 10 shows the relationships among the data in Table 3. The *X*-axis refers to acceleration and sound pressure levels, and the *Y*-axis refers to the square root of the harvested energy and sound pressure levels. All values are represented in decibels. As shown in the plots, data on acceleration, sound pressure level, and harvested energy appear to be linearly related. Calculated correlation coefficients between acceleration and sound pressure, acceleration and harvested electric energy, and sound pressure and harvested electric energy are over 0.976, as shown in Table 4. This reveals that harvested energy, floor vibrations, and sound pressure level are strongly correlated with each other, and a threshold for harvested energy can be employed for detecting floor vibrations and impact sound.

## 4. Demonstration of the Self-Powered Wireless Sensor for Floor Vibration Detection

**Figure 11:**
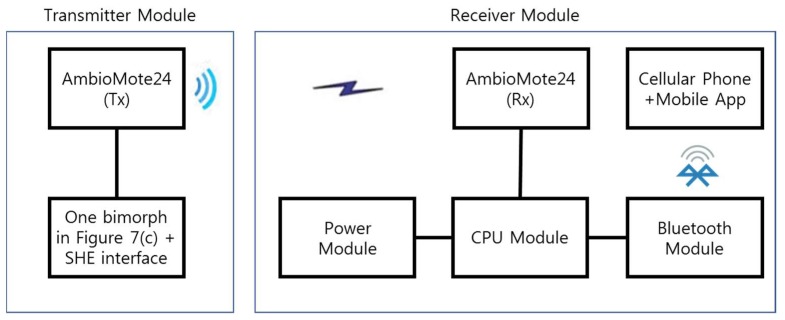
Configuration of the self-powered wireless sensor.

For feasibility demonstration, a self-powered wireless sensor and mobile application software are developed. Figure 11 shows the configuration of the sensor. The transmitter module is composed of one tuned bimorph in Figure 5b and the data transmitting board, AmbioMote24(Tx) [23]. The receiver module has a CPU module, a Bluetooth module, AmbioMote24(Rx), and a power module. Table 5 shows the specifications of each module. When the energy harvester charges electric energy over a certain threshold due to abnormal floor vibrations, charged electric energy powers AmbioMote24(Tx) and sends a datum to AmbioMote24(Rx). Next, the CPU module activates the Bluetooth module, and it communicates with a cellular phone, and the alarm signal is displayed in the mobile application software.

**Figure 12:**
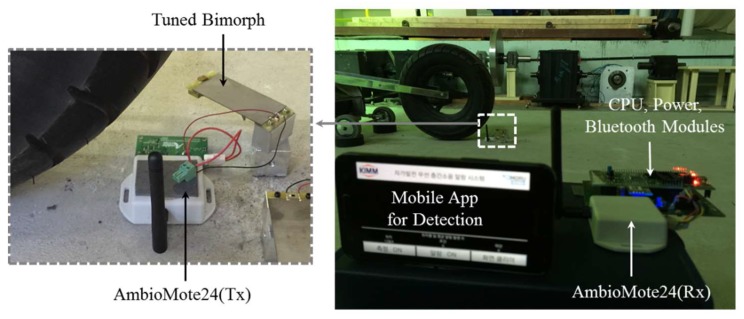
Test setup for feasibility demonstration.

Figure 12 shows the test setup for the feasibility demonstration. The transmitter module is installed on a bare slab, and the receiver module is located about 2 m away from the center of the slab. A cellular phone is set by the receiver module. When impacting the slab with a bang machine and an impact ball, the number of alarm signals displayed in the mobile application software is monitored. Table 6 summarizes the number of data transmissions caused by slab impacts. The harvested electric energy from a one-time impact of a bang machine at the center of the slab can send seven alarm signals to a smartphone [25]. Here, one detection signal means one data transmission from the transmitter module to the receiver module. The electric energy used for one-time data transmission is about 11 μJ (≈78.11 μJ/7 transmissions in case a 47 μF capacitor is used). Differently, three successive impacts using an impact ball can send four alarm signals [25]. This is because the wireless transmitter, AmbioMote24, initiates transmission when the capacitor voltage is charged up to 5.3 V [10]. By using measured harvested energy in Table 3, we can check that one impact with the bang machine at the locations ②~⑤ can transmit one signal. This is because the bimorph can harvest about 11.71 μJ~12.38 μJ. Similarly, we estimate that 23 impacts with the impact ball at locations ②~⑤ can transmit four signals. Based on the demonstration, it is confirmed that the cantilever-type piezoelectric harvester with the SEH interface circuit can generate enough electric energy to send wireless signals with one impact of standard heavyweight impact sources. If the vibration level of an interested abnormal condition is lower than the vibration induced by the standard heavyweight impact sources used, the harvestable energy will be smaller than in Table 3. For example, as described in [26], the impact forces of walking adults, running children, and jumping children are about 500 N, 1000 N, and 3000 N, respectively. As shown in Table 1, the impact force of the bang machine is about 4000 N. Based on the assumption that the acceleration amplitude of a slab is proportional to the impact force and to the square root of harvested energy, the harvested energy of a walking adult (impact location ①) can be roughly estimated as 1.22 μJ (78.11(μJ) × (500(N)/4000(N))^2^). Similarly, the harvested energies of running children and jumping children can be estimated as 4.88 μJ and 43.94 μJ, respectively (impact location ①). The applications with higher thresholds than the harvested energies can be solved with two system modifications: (1) the amount of harvested energy can be easily multiplied by connecting a single energy harvester in parallel; and (2) the electric circuit design can be enhanced to minimize quiescent power by considering the sporadic nature of floor impacts. We believe a study on the suitable thresholds for different applications should also be considered as important future work.

## 5. Conclusions

For the realization of a smart building, Internet of Things (IoT) applications with conventional acceleration sensors have been widely researched for implementing building automation with energy management technologies, and for resolving social issues, such as inter-floor noise-related disputes in apartments and the solitary death of an elderly person living alone. However, the installation of conventional accelerometers is sometimes controversial due to privacy invasion, especially if it is developed as a wireless system. To tackle this issue, this paper proposes a self-powered wireless sensor using a threshold-based method for the detection of floor vibrations. The vibration levels of the slab in the testbed are first measured by impacting the slab with the bang machine and the impact ball. By considering the vibration levels of the slab, the piezoelectric energy harvester is designed to generate electric energy over a certain threshold. A strong correlation among floor impact sound, slab vibrations, and electric energy harvested is revealed through the correlation study, and it is revealed that harvested electric energy can be used as a threshold to predict floor vibrations and the corresponding impact sound in buildings or apartments. Finally, the prototype of the self-powered wireless sensor is developed and its applicability is demonstrated. It is confirmed that the bimorph in Figure 5b with an SHE interface circuit can generate enough electric energy to send wireless signals by impacting with standard heavyweight impact sources.

Due to the characteristics of the sensor—indirect measurement and less invasion of privacy—possible applications will be IoT systems to support a mediation process for inter-floor noise-related disputes in apartments with objective data on human-induced floor vibrations, or to detect the solitary death of an elderly person living alone by monitoring unusual patterns in floor vibrations. In addition, the benefits of using energy harvesters—easy installation and no maintenance costs to supply power in a transmitter—can expand the range of applications with technical improvements in designing vibration-based energy harvesting devices. Regarding future work, designing optimal electric circuits in consideration of floor vibration characteristics will be one of our research topics. For example, the development of electric circuits is required to minimize quiescent power by considering the sporadic nature of floor impacts [22], or to maximize harvestable power by using a decaying sinusoidal wave with a short duration, as shown in Figure 3. In addition, from a reliability point of view, various uncertainties in designing energy harvesters and in apartment environments, such as variation in the natural frequencies of slabs, should be considered [27]. Techniques for designing broadband energy harvesters may be a possible solution to these uncertainty problems. Finally, alarm thresholds and detection algorithms should be studied further.

## Figures and Tables

**Table 1:** Dynamic characteristics of the bare slab.

Impact Source		Natural Frequency of Slab	Acceleration (Peak to Peak)	Time Duration of Slab Vibration
Bang machine		24.8 Hz	−3.0 m/s^2^ ~4.0 m/s^2^	≈0.8 s
Drop height	0.85 m			
Tire pressure	240 kPa			
Impact force (peak)	≈4000 N			
Time duration	≈20 ms			
Impact ball		24.8 Hz	−1.17 m/s^2^ ~1.55 m/s^2^	≈0.6 s
Drop height	1 m			
Impact force (peak)	≈1500 N			
Time duration	≈20 ms			

**Table 2:** Specification of the bimorph specimen [24].

Specification	Value
Model number	D220-A4-503YB
Piezo material	PZT-5A
Size of a base specimen	63.5 × 31.8 mm
Active length of the bimorph (Figure 5a)	57.15 mm
Weight	10.4 gram
PZT thickness	0.019 mm
Brass thickness	0.013 mm
Capacitance	232 μF
Natural frequency (without mass)	52 Hz

**Table 3:** Summary of test results.

Impact Machine	Impact Location	Distance from Accelerometer & Bimorph	Harvested Energy (Average of Two Bimorphs) (μJ)	Square Root of Harvested Energy (dB, Ref.:1 μJ))	Acceleration at 25 Hz (dB, Ref.: 10^−3^ cm/s^2^)	SPL at 25 Hz (dB, Ref.: 20 μPa)
Impact Ball	①	27 cm	10.75	10.31	83.50	78.13
Bang Machine	①	27 cm	78.11	18.93	91.29	85.62
Impact Ball	①	151.7 cm	0.95	−0.22	75.46	69.77
Bang Machine	①	151.7 cm	17.29	12.38	85.59	79.30
Impact Ball	①	151.7 cm	1.69	2.27	77.23	71.73
Bang Machine	①	151.7 cm	14.83	11.71	86.36	79.88
Impact Ball	①	151.7 cm	1.28	1.09	75.65	72.70
Bang Machine	①	151.7 cm	17.20	12.36	84.87	81.25
Impact Ball	①	151.7 cm	1.82	2.60	76.43	73.12
Bang Machine	①	151.7 cm	16.77	12.25	86.05	80.65

**Table 4:** Calculated correlation coefficient.

Factors	Correlation Coefficient
Acceleration vs. Sound Pressure Level	0.976
Acceleration vs. Harvested Energy	0.993
Sound Pressure Level vs. Harvested Energy	0.989

**Table 5:** Specifications of the self-powered wireless sensor.

Module	Main Specifications
AmbioMote24	AmbioSYSTEMS AmbioMote24-A
	Frequency band: 2.4 GHz
	ADC Convert resolution: 10 bit
	Capacitance: 0.5 μ
	Communication distance: up to 80 m
	Data transmission rate(Tx): 10 Hz
CPU Module	STMicroelectronics STM32L152RCT6
	Clock speed: max. 32 MHz
	Data bus width: 32 bit
	Memory: 256 KB, 32 KB RAM, 8 KB ROM
Bluetooth Module	Firmtech FB155BC(SPP + HID)
	Bluetooth Version: 2.1 (2.4 GHz ISM Band)
	Communication distance: 10 m

**Table 6:** Impact condition and the number of data transmissions.

Impact Source	Impact Location	Number of Impacts	Number of Signals Transmitted
Bang machine	①	1	7
Impact ball	①	3	4
